# ‘Recovery’ in the Real World: Service User Experiences of Mental Health Service Use and Recommendations for Change 20 Years on from a First Episode Psychosis

**DOI:** 10.1007/s10488-018-0851-4

**Published:** 2018-02-06

**Authors:** Donal O’Keeffe, Ann Sheridan, Aine Kelly, Roisin Doyle, Kevin Madigan, Elizabeth Lawlor, Mary Clarke

**Affiliations:** 1 DETECT Early Intervention in Psychosis Service, Dublin, Ireland; 2 School of Nursing and Midwifery, Trinity College Dublin, Dublin, Ireland; 3 School of Nursing, Midwifery, and Health Systems, University College Dublin, Dublin, Ireland; 4 Saint John of God Hospitaller Services, Dublin, Ireland; 5 Saint John of God Community Services, Dublin, Ireland; 6 School of Postgraduate Studies, Faculty of Medicine and Health Sciences, Royal College of Surgeons in Ireland, Dublin, Ireland; 7 School of Medicine and Medical Science, University College Dublin, Dublin, Ireland

**Keywords:** Recovery, Mental Health Services, Policy, Legislation, Psychotic disorders, Qualitative research

## Abstract

Little is known about how recovery oriented policy and legislative changes influence service users’ perceptions of mental health care over time. Although the recovery approach is endorsed in many countries, qualitative research examining its impact on service use experiences has been lacking. This study aimed to explore this impact as well as experiences of service utilisation and suggestions for change with people diagnosed with a First Episode Psychosis between 1995 and 1999. Participants had used services during the 10 year period prior to, and 10 years post, policy and legislative shifts to the recovery approach. Semi-structured interviews were conducted with 10 participants who met criteria for ‘full functional recovery’ and 10 who did not. Data were analysed using Thematic Networks Analysis to develop Basic, Organising, and Global Themes. Over time, recovered participants perceived an improvement in service quality through the ‘humanising’ of treatment and non-recovered participants experienced their responsibility in recovery being recognised, but felt abandoned to the recovery approach. Findings suggest the importance of viewing service users as demonstrating *personhood* and having *societal value*; examining the personal meaning of psychotic experiences; and matching expectations with what services can feasibly provide. The implementation and the principal tenets of the recovery approach warrant further investigation.

## Introduction

The recovery approach to service delivery, the recovery model, or simply ‘Recovery’ is the cornerstone of mental health policy in many Western countries (Le Boutillier et al. 2011) and recently has extended to Asia and Africa (Ministry of Health and Family Welfare 2014; Department of Health 2013). ‘Recovery’ based services seek to integrate the input of, and achieve the outcomes prioritised by, service users and their family in order to optimise mental health treatment. These tenets of ‘Recovery’ include: (i) practitioners holding optimism for recovery for all and respecting each service user’s uniqueness, personhood, expertise, and the personal meaning of their experience; (ii) services contributing to tackling the social, political, and economic barriers to citizenship, social integration, and inclusion; (iii) psychiatric assessments interpreting perceived deficits, pathology, and symptoms within a strengths and resilience framework; (iv) systems emphasising empowerment, collaborative decision making, self-determination, choice, and risk-taking in individualised, person-centred, recovery planning; (v) health care organisations prioritising access, engagement, continuity of care, and the incorporation of user led services; and (vi) discourse among practitioners reflecting a multiplicity of biological, psychological, social, and spiritual perspectives on the aetiology of ‘mental illness’ (Commonwealth of Australia 2013; Higgins 2008; Davidson et al. 2009).

Quantitative evidence supporting ‘Recovery’ is emerging (Slade and Longden 2015). Studies have demonstrated the efficacy of case management emphasising recovery principles (Gelkopf et al. 2016), service user managed services (Greenfield et al. 2008), and specific ‘Recovery’ based interventions such as peer support and self-management (Davidson et al. 2012; Cook et al. 2012). However, how efforts to implement the recovery approach, in its totality, influence the service user experience is uncertain. Central to the recovery model is the concept of recovery itself. Services are asked to focus their energies and resources on prioritising service user defined (i.e. personal) recovery over clinical recovery (Slade 2009). Empirical data indicate that these are markedly divergent yet overlapping constructs (Van Eck et al. 2017; Macpherson et al. 2016). More research is required to further operationalize the recovery concept and strengthen the evidence base for the effects, applicability, and acceptability of the recovery approach and its discrete components.

Internationally, deinstitutionalisation, the adoption of community based psychiatry, and the promotion of the recovery approach have led to significant changes in policy and service provision. In Ireland, this transformation was instantiated by (i) the Mental Health Act (Irish Statute Book 2001) (legislation fully implemented in 2006 to safeguard service user rights and ensure care standards) and (ii) A Vision for Change (Department of Health and Children 2006) (a national mental health policy underpinned by a commitment to service user and family/carer involvement, recovery optimism, access and engagement, continuity of care, social inclusion, and mental health promotion). Policy/legislation changes represent a pivotal aspect of the context of service use; therefore any exploration of the service user experience must take these into account. Since 2006, to realise the recovery approach, the Irish Mental Health Commission designed an audit tool to assess implementation (Higgins 2008) and the Irish health service launched *Advancing Recovery in Ireland* to support the development of local stakeholder groups in enhancing ‘Recovery’ practices (e.g. recovery principles training, recovery colleges, and peer support). Recently, a national office to promote service user, family member, and carer engagement in the design, implementation, and evaluation of services has been established. However, in 2017, the uptake of a recovery orientation in services remains slow and patchy in parts of Ireland.

Broadly speaking, mental health service users have reported relatively high satisfaction with inpatient and community based services (Bø et al. 2016; Boydell et al. 2012; Ruggeri et al. 2007). However, concern has been expressed regarding the validity of such evaluations and the limited variability in ratings. These quantitative studies fail to acknowledge the equivocality of ‘consumer satisfaction’, the mixed relationship between satisfaction and clinical outcomes, and the influence of social desirability (Aarons et al. 2010). Quantifying satisfaction in the absence of developing an understanding of service user expectations of, and approaches to, evaluating services may perpetuate the status quo. Ratings fail to explain (dis)satisfaction (Martin et al. 2003), may not mirror experience (Price 2014), and provide limited guidance for quality improvement (Garland et al. 2010).

While service user involvement is recognised by providers and policy makers as imperative, genuine commitment to it has been questioned (Bennetts et al. 2011). There is a risk that the recovery approach may become subverted to serve purposes that are not service user defined (Beresford 2015). The challenge for services is to authentically acknowledge the value of service user input and to meaningfully integrate it to enhance service provision. One way of achieving this is to explore service user perspectives of treatment and change over time and to utilise them to drive change. It is also necessary to capture the feedback of people across the clinical recovery continuum as, to date, ‘recovered’ service users may have had their viewpoints more widely conveyed.

Qualitative research can help develop an understanding of service use in context, determine how interventions are experienced and implemented, and examine the limitations of and barriers to evidence based medicine. Exploring service user perspectives can enable a nuanced appraisal of the effects of policy/legislation changes. This is important in order to establish if these changes improve the quality of mental health services and the service use experiences of those who utilise them. Service users may hold less positive views on recovery orientation than clinical team leaders (Leamy et al. 2016). Qualitative methods can help elucidate why this might be. This research can inform service developments so they meaningfully engage with service users and support their personal resourcefulness, self-defined recovery goals, and capacity to recover.

### Aims

The primary aims of this study were to explore from the perspectives of people diagnosed with a First Episode Psychosis (FEP) 20 years ago: (i) their experience of community based and inpatient mental health service use over 20 years; (ii) their perceptions of change in service delivery over time; and (iii) their recommendations for service improvement. This is a unique sample of people who had 10 years’ experience of mental health services prior to, and 10 years post, policy and legislative shifts to the recovery approach. Participants represent a diverse breadth of psychotic experiences; having embarked a divergent range of recovery trajectories following initial FEP diagnosis. A secondary aim was to examine the impact of recovery oriented policy/legislation on service use experiences. Although these may be shaped by multiple factors, Irish services did respond to policy/legislation modification by endeavouring to implement the recovery approach. Our sample offered a novel opportunity to retrospectively understand how participants experienced this change.

## Methodology

### Design, Context, and Ethical Considerations

This paper reports on the qualitative arm of the iHOPE-20 (Irish Health Outcomes in Psychosis Evaluation—20 year follow up) study. This is a prospective 20 year FEP follow up study conducted between 2014 and 2017, in Ireland. All participants received a dedicated assessment approximately 20 years ago when they had first contact with a private/public health care organisation based in a Dublin catchment area where they resided (Clarke et al. 2006). At time of interview, most continued to live in Dublin, with some residing in different parts of Ireland. Participants received varying degrees of support in mental health settings over the 20 years in Ireland and other countries and some lived in service managed accommodation. Over the 20 year period, treatment modalities and support services available within the research site catchment area included outpatient psychiatric review, inpatient hospitalisation, an acute day hospital, medication management, community mental health nurse home visits, residential rehabilitation, day centres, occupational therapy, and a range of psychological interventions, social work, and family supports. In Ireland, recovery oriented policy/legislation came into effect in 2006 and services were adapted in response to these to promote personal recovery in addition to clinical recovery. Anonymity was managed by pseudonymising/redacting all individual, group, and service/place names from transcripts. A service user contributed to study design. The study was approved by the local ethics committee.

### Sampling and Recruitment

Twenty service users were purposefully sampled from a representative epidemiological cohort of 171 individuals diagnosed with a FEP between 1995 and 1999 using the SCID-IV (Structured clinical interview for DSM-IV axis I disorders; First et al. 1995). We define FEP as “first presentation with acute psychotic symptoms to any psychotic service for patients who, if they had been prescribed antipsychotic medication prior to presentation, had been receiving such treatment for no more than 30 days” (Hill et al. 2012, p. 216). Exclusion criteria were: people with an intellectual disability/an organic disorder and people unable to provide informed consent. Quantitative data were used to sample participants by clinical recovery status. To select our sample, we began with the 80 individuals who had participated in the quantitative arm and completed a 20 year follow up quantitative assessment of recovery, remission, physical health, and service use. Five people declined to be contacted for the qualitative component; reasons given included not having the time to participate, being tired of research, and not wanting to give any further information. Of those that were willing to hear from us again (n = 75), we reviewed age and gender demographics and applied a clinical recovery criteria (‘full functional recovery’ [Alvarez-Jimenez et al. 2012]) to recruit a sample that would provide a continuum of experiences from multiple perspectives. We selected 10 people who met full functional recovery criteria and 10 who did not. All people subsequently approached agreed to participate.

Full functional recovery comprises remission, functional status, and vocational status recovery. Excluding the 6-month duration component, the remission criteria advocated by Andreasen et al. (2005) was used to assess symptomatic remission. Remission of positive and negative symptoms was defined as a score of ≥ 2 on 8 Positive and Negative Syndrome Scale items (Kay et al. 1987): delusions; unusual thought content; hallucinatory behaviour; conceptual disorganization; mannerisms/posturing; blunted affect; social withdrawal; and lack of spontaneity. Functional and vocational status recovery were defined as a score of ≥ 4 on 4 Quality of Life Scale questions (Heinrichs et al. 1984): appropriate interpersonal relationships with people outside of family; adequate vocational functioning (being in paid employment, attending school, or performing homemaker role effectively); adequate achievement in role adopted; and basic living task engagement. Although the ‘full functional recovery’ concept could be considered at odds with the personal recovery and process emphases of the recovery approach, we considered its utilisation the most appropriate strategy to attain the broad range of viewpoints required. Some service users may not identify with the concept of personal recovery or see personal recovery as an outcome. We believed that the objectivity granted by a clinical recovery cut off would clearly delineate groups and enhance the rigour of our study. This strategy allowed for an exploration of the nuances that arise within and between clinical recovery status groups. The sample size (N = 20) was deemed sufficient to answer our research questions, to promote transferability to other contexts, and to explore patterns of similarity and difference.

Participants were recruited to the quantitative arm using registered letters, phone calls, and clinician contact (mental health practitioners and General Practitioners). Following participation in the quantitative phase, cohort members were asked if they were agreeable to contact for the qualitative component. If willing, cohort members were then met by DOK to obtain written informed consent.

### Data Collection and Analysis

Data collection involved 20 semi-structured interviews, lasting 22–90 min, conducted in places of convenience for participants (i.e. mental health services, hotels, participant’s homes). Semi-structured interviews were selected to capture the diversity and complexity of participants’ experiences and to shed light on their context. Interviews were guided by a topic guide that covered three general areas of interest (Table 1). The guide was developed by a multidisciplinary working group which included a service user with experience of psychosis. Its content was derived from the group’s personal, clinical, and research expertise and a comprehensive literature search. Participants were questioned about their experiences from first contact with mental health services to the date of the interview. The first 2 interviews (1 in each group) were completed as pilot interviews to trial and refine questions and prompts in order to standardise the guide. Following the pilot interviews, questions were modified to reflect a focus on participants’ overall experience of service use (not just their predominant memories) and participants’ experience of change or inertia (in addition to asking if change was perceived). DOK performed 18 interviews, AS completed 2. Both interviewers were experienced qualitative researchers who were unknown to participants prior to participation. All interviews were audio-recorded and transcribed for analysis.

**Table 1:** Summary of interview topic guide

*Experience of mental health service use* ^a^
How have you experienced mental health services over the last 20 years?
What do you think are the three major strengths of the existing mental health services?
What do you think are their three major weaknesses?
*Perceptions of change in service delivery over time* ^b^
Have you experienced any changes in mental health services over the last 20 years?
If so, how did you experience these changes?
If so, have these changes influenced your recovery?
If not, has the lack of change influenced your recovery?
*Recommendations for service improvement* ^c^
What three changes would you make in mental health services to make them more supportive of recovery?

^a^Was it positive, negative, or neutral?

^b^Did it improve, get worse, or stay the same?

^c^What improvements you would suggest?

Thematic Networks Analysis (TNA) was selected as a research method as it provided a practical, robust, and efficient technique for sensitive, insightful, and rich exploration of the structures and patterns within our dataset (Attride-Stirling 2001). TNA aims to explore the understanding of an issue or the meaning of a concept in peoples’ lives rather than integrating and reconciling different perspectives. Analysis was exploratory and inductive, with data being analysed at a semantic level. The TNA approach is based on the principles and epistemological/ontological assumptions of Argumentation Theory (Toulmin 1958) which asserts that all findings, while reasonable, are not absolute as multiple realities exist. Analysis proceeded across six steps: (i) coding material; (ii) identifying themes; (iii) constructing thematic networks; (iv) describing and exploring networks; (v) summarising networks; and (vi) interpreting patterns. Basic Themes were developed from initial codes, grouped into Organising Themes (abstract principles), and integrated to form Global Themes (principal metaphors). Themes were then displayed in web like maps. DOK performed the initial analysis which was then validated through consultation with AS, AK, and MC. Data were coded and analysed using NVivo 11 to enhance flexibility, thoroughness, validity, and rigour. Data analysis was directed by ‘information power’, an aspect of internal validity based on the contribution of original knowledge obtained from analysis (Malterud et al. 2016). Information power was deemed robust as the study aim was broad, the specificity of the sample was dense, an extensive theoretical background was used in data interpretation, cross case analysis was utilised, and dialogue was strong.

## Results

**Fig. 1:**
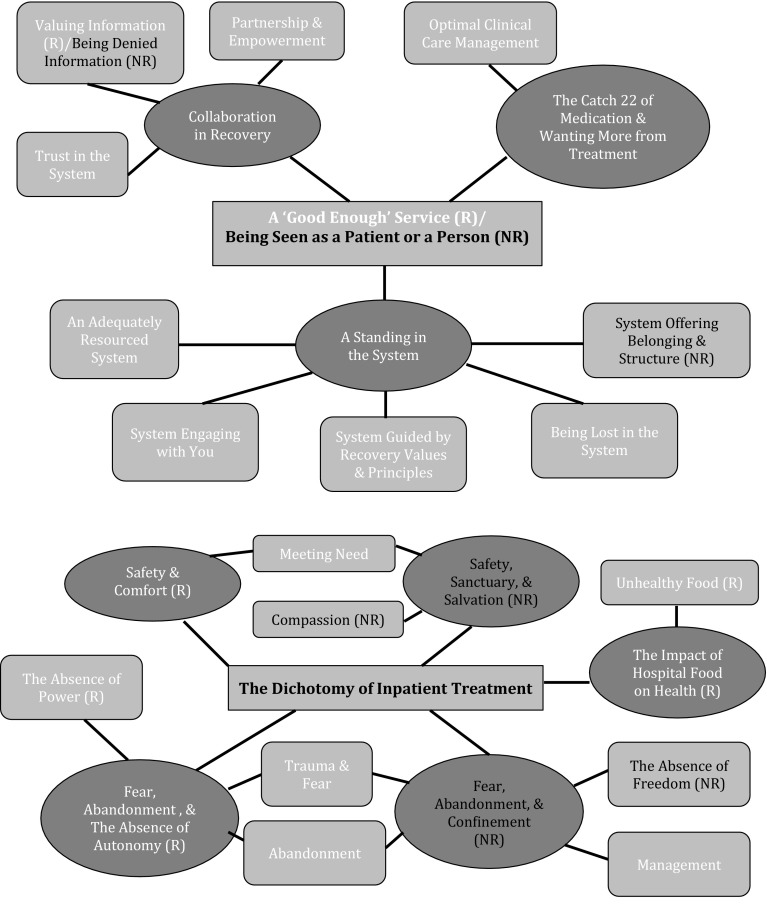
Thematic networks of experience of service use: community based and inpatient. Key: *R* Recovered Group, *NR* Non Recovered Group. Colour coding: *Red* Global Theme, *Blue* Organisational Theme, *Purple* Basic Theme. (Color figure online)

**Fig. 2:**
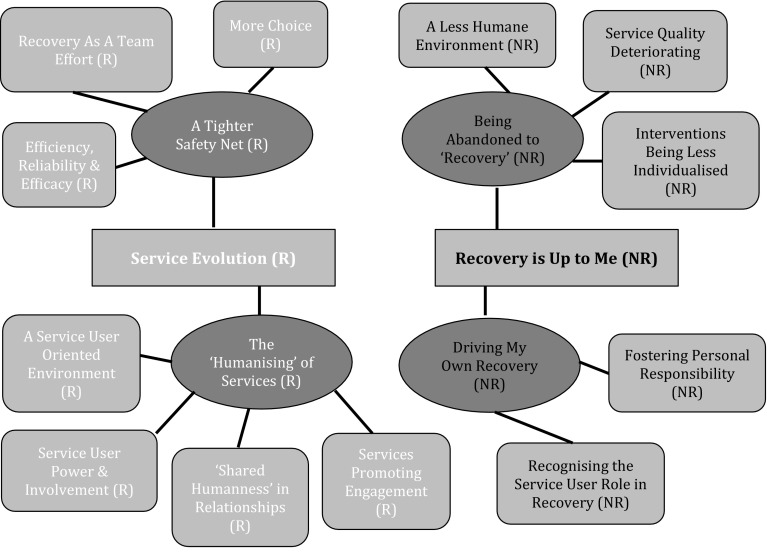
Thematic networks of experience of change over time. Key: *R* Recovered Group, *NR* Non Recovered Group. Colour coding: *Red* Global Theme, *Blue* Organisational Theme, *Purple* Basic Theme. (Color figure online)

**Fig. 3:**
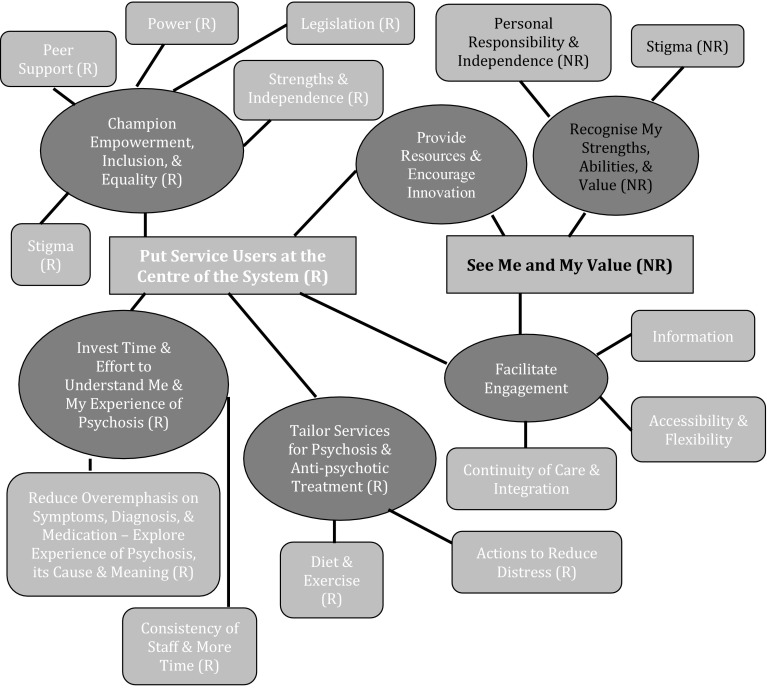
Thematic networks of recommendations for service change. Key: *R* Recovered Group, *NR* Non Recovered Group. Colour coding: *Red* Global Theme, *Blue* Organisational Theme, *Purple* Basic Theme. (Color figure online)

Characteristics of the sample are displayed in Table 2. Differences between both groups reflect their clinical recovery status. Thematic networks for each question are summarised in Figs. 1, 2, and 3.

**Table 2:** Demographic characteristics and diagnoses of study sample

Characteristic [M(SD)/*n* (%)]	Recovered	Non-recovered	Entire sample
Age in years at time of interview	40.5 (7.26)	46.6 (7.76)	44.55 (7.25)
Ethnicity			
Caucasian	10 (100%)	10 (100%)	20 (100%)
Gender			
Male	6 (60%)	6 (60%)	12 (60%)
Female	4 (40%)	4 (40%)	8 (40%)
Baseline SCID-IV diagnosis (1995–1999)			
Schizophrenia	3 (30%)	6 (60%)	9 (45%)
Schizophreniform disorder	0 (0%)	1 (10%)	1 (5%)
Delusional disorder	1 (10%)	1 (10%)	2 (10%)
Bipolar disorder with psychotic features	5 (50%)	1 (10%)	6 (30%)
Major depression with psychotic features	1 (10%)	1 (10%)	2 (10%)
Employment status			
Full-time employment	6 (60%)	0 (0%)	6 (30%)
Part-time employment (≤ 30 h per week)	2 (20%)	1 (10%)	4 (20%)
Full-time student (≥ 30 h per week)	1 (10%)	0 (0%)	1 (5%)
Unemployed	0 (0%)	9 (90%)	9 (45%)
Home-maker	1 (10%)	0 (0%)	1 (5%)
Relationship status			
Single	4 (40%)	7 (70%)	11 (55%)
Married	5 (50%)	1 (10%)	6 (30%)
Engaged	0 (0%)	1 (10%)	1 (5%)
Living with partner	1 (10%)	0 (0%)	1 (5%)
Separated/divorced	0 (0%)	1 (10%)	1 (5%)
Highest level of education attained			
Primary level	0 (0%)	1 (10%)	1 (5%)
Secondary level or equivalent	2 (20%)	2 (20%)	4 (20%)
Specific vocational training	0 (0%)	3 (30%)	3 (15%)
Third level certificate	0 (0%)	1 (10%)	1 (5%)
Third level diploma/degree	5 (50%)	2 (20%)	7 (35%)
Third level postgraduate degree	3 (30%)	1 (10%)	4 (20%)

### Experiences of Service Use

While Organising Themes identified for community based service use for both groups were identical, nuances found among Basic Themes led to two separate Global Themes: *A ‘Good Enough’ Service* (recovered group) and *Being Seen as A Patient or A Person* (non-recovered group). The former embodied the overarching belief that services were satisfactory. There was gratitude for the role of treatment in recovery but also an awareness of significant shortcomings. The latter was defined by a continuum of experiences from (i) having relationships with practitioners based on a ‘shared humanness’ which supported personhood to (ii) being treated with an absence of respect, sensitivity, and empathy, and feeling reduced to one’s symptomology. Margaret (non-recovered, female, 43) describes her personhood being nurtured:

They taught you that you are a person. They taught you like – ‘Well this is what I’m doing’. Like you know, when they go on their holidays and you go – ‘Well how did you get on, on your holidays?’ Just that type... not become their friend.

Experiencing *Collaboration in Treatment* came from shared decision making, peer support, and self-management. While recovered participants valued information important to their recovery (e.g. an explanation of the tribunal process), non-recovered participants believed they were denied it. Both groups reported that at times they struggled to develop trust in the system as they felt they were unable to form relationships with its staff. Participants described how retelling their story to different practitioners hindered them moving on from their past. Trust was fostered when practitioners personally invested in them and expressed a respectful curiosity about their lives. The connection, support, and safety of these relationships acted as stepping stones in recovery.

Both groups detailed how they grappled with adhering to antipsychotic medication (*The Catch 22 of Medication*). Sacrificing one’s energy, self-image, sexuality, and social functioning to get control over, and experience respite from, the positive symptoms of psychosis. Most experienced barriers to identifying and maintaining the right medication and dose (e.g. its prohibitive cost). Both groups *Wanted More from Treatment*. They perceived an absence of depth in assessment and craved a comprehensive psychological analysis to get to the root of their problems. A lack of interest in understanding their experience of psychosis or the content of their symptoms was felt.

All participants experienced the presence or longing for *A Standing in the System* (i.e. their status to be acknowledged with resources, engagement efforts, and actions that demonstrated egalitarianism, respect, guidance, and belonging). A recovery ideology in services was both present (e.g. having strengths emphasised) and absent (e.g. being treated as children). Everyone interviewed portrayed a system that was stretched and overburdened and felt they had unmet mental health needs (e.g. no trauma interventions). This, in some cases, led to the perception that practitioners were ignoring their problems. The system actively sought to engage them (by being accessible, flexible, and integrated) or inadvertently caused them to disengage from treatment. Some people felt lost in the system, recovering outside of it entirely by outsourcing external help. Day centres and vocational courses offered structure and belonging to non-recovered participants.

For both groups, reflecting on inpatient service use involved integrating dissonance: *The Dichotomy of Inpatient Treatment*. They perceived hospital as a *Safe* haven; protecting them and monitoring their health. Recovered participants emphasised the *Comfort* of the setting; the non-recovered, the *Sanctuary and Salvation* it provided. Lesley (non-recovered, female, 49) describes being protected from harm:

Suddenly I was getting the right attention but I felt... I suppose I was in the safest place. If I was out on the street wandering around, God knows what would have happened.

Both groups described inpatient treatment as traumatising due to exposure to coercion, forced treatment, aggression, substance misuse, self-harm, thought disorder, and suicidal ideation. Participants were fearful of other service users, the tribunal process, the police, and multi-disciplinary team (MDT) members. A sense of *Abandonment* was borne by both; some people, like Chris (recovered, male, 37), felt ignored and discarded:

You know, it’s mind-numbing at times that you are left alone to your own devices and all you got are your devices.

Recovered participants communicated frustration at the lack of their *Autonomy* and power in inpatient care. Non-recovered participants felt *Confined* and identified management issues (e.g. no differentiation between first episode service users and others).

### Perceptions of Change in Service Delivery over Time

For recovered participants, these were encompassed by the Global Theme of *Service Evolution*, which incorporated the *‘Humanising’ of Services*. Practitioners were now personally invested in their recovery, proactively checked in, and were ‘in tune’ with them. Thomas (recovered, male, 47) describes his experience:

He was my first… contact of a human being who wasn’t hiding behind a veil of the professionalism of ‘I am a psychiatrist’. He used to ask me about my car and he used to look out the window at my car and his car… he was really interested in me as a person, just as much as he was at managing my symptoms.

*A Tighter Safety Net* was now present. This meant increased choice, a more efficient response to need, and enhanced intervention reliability and efficacy. In time, recovery had become a team effort; participants now shared responsibility for it with their MDT and community.

For non-recovered participants, the Global Theme of *Recovery is Up to Me* incorporated *Being Abandoned to ‘Recovery’*. They perceived a reduction in quality of care, the absence of outreach, less comprehensive assessments, and less interaction with and attention from practitioners. Some did not identify with the concept of recovery and felt deserted by services. For them, the recovery approach was seen as the removal of structure within and the dumbing down of programmes. Gabriel (non-recovered, male, 57) details how he laments the direction provided by services in the 1990s/early 2000’s:

Well I think someone was talking there that they have moved from a medical model to a recovery model in [day centre], so everything is a bit freer and easier. It is a bit of a lowest common denominator thing, the discipline is terrible in this place, you know, and there might have been regimentation before, but it is a case of lack of conviction. There is no one saying, ‘Take this, it will do you good’ or ‘This is it’.

These participants believed that within the modern system, there was no acknowledgment of how there are different ways to recover. Some noted a reduction in empathy and a more anxiety provoking atmosphere. This Global Theme also integrated *Driving One’s Own Recovery*: being recognised as an active, autonomous, responsible agent in recovery.

### Recommendations for Service Change

Some Organising Themes relating to resources, innovation, and engagement were shared between both groups (e.g. supplying an early intervention nurse in accident and emergency departments). Both groups desired providers to confront stigma to normalise psychosis and enhance social inclusion. For the recovered group, recommendations were encapsulated by *Put Service Users at the Centre of the System* (the request to design treatment around their needs, rather than the system needs of efficiency and responsibility boundaries). Doing this involves power sharing, acknowledging service user capacity, and implementing further legalisation changes (e.g. enshrining the right to be given a rationale for hospitalisation). Some themes were psychosis specific. Ruth (recovered, female, 56) suggests reducing psychosis related distress by being introduced to each practitioner separately before attending MDT meetings:

So I would have had a one to one with the psychiatrist... Because you are quite brittle, all these people, you know and they are all firing questions at you. That is the only thing, I know it was good that you had all these different expertise and professionals, but I would have preferred it one to one.

Recovered participants advised the availability of nutritious food and an emphasis on diet and exercise to counteract the side effects of anti-psychotics. This group wanted to examine their experience of psychosis and why they were distressed, but had no space in treatment to do so. They wanted continuity of their most valued relationships with practitioners.

For the non-recovered group, their recommendations were summed up by the Global Theme *See Me and My Value*. Participants sought interactions with service providers that promoted equality and self-determinism (e.g. sanctioning them for non-attendance). These recommendations were a response to services ‘mollycoddling’ them and cultivating dependence.

## Discussion

Findings illuminate the real world impact of policy and legislation changes on the lives of service users. While experiences are comparable between groups, perceptions of change over time are distinct, and recommendations for improvement are both similar and discrete. People who recovered clinically described predominant satisfaction; possibly due to them meeting society’s normative definition of recovery at time of interview and recognising the role of services in achieving this outcome. However, they also felt failed by the system at times. Recovered participants wanted a value based service centred on justice, equality, respect, compassion, and empowerment. Generally speaking, they felt they received it; but like previous research, they acknowledged that the system can undermine the capacity of staff to provide it (Williams and Tufford 2012). Funding problems and legal, clinical, and risk issues may pose key challenges to recovery orientation.

The quest for self-determination and recognition is fundamental to service user involvement in rehabilitation (Petersen et al. 2012), therefore it follows that non-recovered participants understood their community based experience in terms of what they were identified as. They aspired to be seen as demonstrating *personhood* and having *societal value*. This group desired to be acknowledged as psychological, social, sexual, spiritual, and physical beings; who require understanding, need security, exist ‘in context’ (Higgins 2008; Barker 2001); and have value in terms of their current or potential functioning, contribution, and competence. Both concepts are particularly important in recovery in psychosis (Davidson 2011; Mezzina et al. 2006) due to its impact on a person’s sense of the self (Davidson and Strauss 1992) and the recovery pessimism inaccurately associated with it (Hill et al. 2012). Person centred care involves attending primarily to the person rather than to a diagnosis or set of symptoms (Roe 2001) and strengthening continuity in identity, an identity not centred on illness, which may be disrupted by psychosis and system contact.

The task of integrating the contradictory qualities of inpatient care and the dissonance between expectations of treatment and its realities has been reported previously (Fenton et al. 2014; Johansson et al. 2007; Stenhouse 2013). This study confirms the presence of this challenge across clinical recovery status groups. Hospitalisation affords protection from the outside world and a respite from the responsibilities of adult life. The refuge and positive therapeutic milieu of inpatient wards counteracts self-destructiveness (Thomas et al. 2002), helplessness and insecurity (Wood and Pistrang 2004); increasing the likelihood of recovery (Borge and Fagermoen 2008). We identified ‘sanctuary harm’ and ‘sanctuary trauma’ among our sample. These experiences are generated from fearful contact with other service users and iatrogenic induced harm from seclusion, constraint, and coercion (Robins et al. 2005; Frueh et al. 2000; Johnson et al. 2004). The former can arise from witnessing actual aggression but also from accepting the dominant discourse connecting ‘mental illness’ and violence, in the absence of knowledge of others or evidence to the contrary (Stenhouse 2013). While it has been reasoned that psychotic experiences are more traumatic than the coercive measures used to control them (Meyer et al. 1999), findings indicate an element of re-traumatisation by inpatient treatment, compounding psychosis trauma. The complex interplay between constraint and safety we identified may be explained by inpatient units being ‘heterotopias’, where people feel safe because the environment allows expressions of distress without drawing excess attention to it—which paradoxically creates disorder precisely because of this permissiveness (McGrath and Reavey 2013).

Our data suggest a polarisation between both groups regarding policy and legislation change and an orientation to ‘Recovery’. Recovered participants observed that services had become ‘humanised’. This change could reflect the move away from psychiatric reductionism of the medical model, where interactions with practitioners may have been dominated by symptoms, diagnoses, and medication. The desire to be recognised as human in mental health care has been widely reported (Eirksen et al. 2012; Williams and Tufford 2012; Davidson et al. 2008). Acknowledging humanity results in positive service evaluations (Beal et al. 2007) and exceptional practitioners display both professional and human qualities (Barker et al. 1999). Being seen as an ordinary complex individual with problems can alleviate the distress associated with the objectification of diagnostic labelling (Larsen and Terkelsen 2014) and reframe ‘mental illness’ as a human challenge (Stastny and Lehmann 2007).

Outside of recognising their role and responsibility in recovery, non-recovered service users struggled to benefit from recovery orientation; they viewed it as the removal of order from services and practitioners forsaking them. One possible explanation for this is that rather than remaining grounded in a genuine commitment to inclusion, autonomy, empowerment, and human rights, the recovery concept has become professionalised and colonised by practitioners to make services more acceptable and competitive; detaching it from its foundation in service user perspectives. The absence of perceived improvement over time we identified could be attributed to the advent of consumerism within the system in the absence of true partnership or any change in power structures (Hui and Stickley 2007). This risks tokenism as service providers can select the most ‘acquiescent’ or ‘appropriate’ service users to fit into their current structures (Ocloo and Matthews 2016) which can serve to legitimise pre-established plans. The language of ‘Recovery’ may be present in policies and the lexicon of practitioners, but the tenets of the recovery approach absent from the service user experience. ‘Recovery’ can be used to abdicate responsibility from providers, transferring duty from the state to the service user (Davidson et al. 2006).

Findings may also be explained by issues related to the recovery model itself. The absence of consensus on the meaning of recovery may have hampered service users identifying with or relating to it. The recovery approach privileges the Western ideals of individualism and espouses that people must recover in a particular way—by being held personally responsible for treatment decisions and actively participating in their recovery. Such goals may be problematic for people who value the idea of collective responsibility or entirely inaccessible to those whose sense of self is engulfed by symptomology (Poole 2011; Peyser 2001). They may experience marginalisation attending a recovery oriented service, promoting a one size fits all approach. ‘Recovery’ therefore may just be for the recovered. Although a recovery orientation requires recovery principles to be applied democratically (Davidson et al. 2009), our data indicate the need to develop ‘readiness’ in people who find it difficult to utilise them. Ultimately, it requires achieving the balance between a recovery orientation and evidence-based practice, moving the locus of control between treatment provider and receiver, at different time points (Frese et al. 2001). The recovery approach may privilege the most articulate, vocal, active (i.e. ‘recovered’) service users as they are more likely than others to influence policy and guide practice. It is also possible for an individual’s wellbeing to improve without using recovery concepts. Being disenfranchised socially, economically, and politically may make recovery unlikely. The recovery model may ignore context and strain to account for the social determinants of health and the relationship between social inequity and recovery. Gender, class, culture, ethnicity, sexual orientation, and disability all determine the accessibility of recovery principles. While the application of recovery concepts can consider these factors [e.g. (Jacobson and Farah 2012)]; contextual sensitivity is not an inherent quality of the recovery approach. Therefore the uncritical conceptualising of recovery as a universal concept (Lal 2010) and its indiscriminating import into incongruent contexts may be problematic. The pressure and necessity to recover can be understood in the context of neoliberalism—the ideology that emphasises the individual and their economic independence regardless of social circumstances (Morrow 2013). Expecting recovery in all conditions may have contributed to the perceived abandonment reported.

Recovered participants recommended depth in assessment and treatment; for their experience of psychosis to be explored and their ‘personal aetiology’ and the meaning of their psychosis given credence. To date, mental health services have largely been disinterested in the personal meaning of psychosis. Clinical practice traditionally views psychosis as devoid of meaning (Dillon et al. 2014). Primary interventions for psychosis are focused on changing biochemistry, modifying behaviour, and enhancing functioning, despite meaning being fundamental to recovery (Leamy et al. 2011). Non-recovered participants advocated for parity with practitioners in their right to promote their interests, reciprocity in relationships, and recognition of their dignity. Central to this acknowledgment of personhood and humanity was practitioners using appropriate self-disclosure to demonstrate theirs. While the positive consequences of self-disclosure have been documented (Ziv-Beiman 2013), it also risks adverse effects (Ziv-Beiman and Shahar 2016), and therefore requires careful management. Moreover, interpersonal relationships directly account for how treatment is perceived (Gilburt et al. 2008). Restrictions on appointment time and the turnover/rotation of practitioners hinder relationship development.

### Policy, Practice, and Research Implications

Findings provide support for, and potentially detract from, recovery oriented policy. Although their evidence base is emerging, there is a need for wider debate and critique of recovery principles, controlled examinations of the efficacy of applying them, and qualitative investigations of the barriers to their utilisation by service users. It may be advantageous for practitioner training to emphasise engagement, communication, and relationship/trust building. Innovative methods to successfully convey information about rights and treatment could be adopted to address the impact of psychosis on cognition (e.g. smart phone apps, videos, seminars). It is important that service users have at least one MDT member who can provide continuity in their treatment over time. It is vital to match service user expectations with what a service can reasonably deliver and to communicate what aspects of recovery organisations have responsibility for. Future research may benefit from comparing service use in diverse countries with differing degrees of recovery orientation and investigating the trauma experienced in inpatient care in order to minimise it.

### Strengths and Limitations

The breadth and depth of the analysis presented permits a juxtaposition of differing service user perspectives overtime and elucidates what is distinctive about the experience of service use in ‘psychotic illness’. Reflexivity, prolonged analysis engagement, independent cross-checking of coding, and the development of a research process audit trail improved credibility and dependability. We limited the impact of our preconceptions by actively searching for data that undermined initial interpretations. The study had a number of limitations. Firstly, outside of the implementation of ‘Recovery’, a complex array of multi-level factors may have influenced participants’ perceptions of change over time. Since the mid 1990s, Irish services have seen expanded numbers and needs of service users; proliferated by national campaigns to increase the visibility of mental health, reduce the stigma of service use, and enhance help seeking. Service providers have increasingly emphasised value based health care, evidence based practice, and multidisciplinary collaboration. Psychosocial interventions have become more accessible and there has been a shift in how the general public and practitioners regard the priorities and parameters of services. Mental health system culture change took place in the backdrop of wider shifts in Irish society; including the ‘maul’ of the Celtic Tiger (the psychological and resource decimating aftermath of the 2008 economic recession), the decline of religion and tradition, the rise of information/communication technologies, and the influx of multicultural perspectives through immigration. Secondly, caution should be applied when interpreting results as participants reported on experiences up to 20 years old; therefore data may be influenced by recall bias. Thirdly, there was no ethnic variation in our sample and the majority of service use described was of one urban based private/public health care organisation. These factors should be taken into account when assessing transferability. Fourthly, we did not interview service providers about their commitment to service user involvement and perceptions of change over time. This could have shed light on why some of our sample had a negative experience of recovery orientation. Finally, while a service user collaborated with us on this study, service user led research may provide novel insights.

## Conclusion

The optimism, egalitarian nature, and revolutionary spirit of the recovery approach have improved the lives of service users who have been able and willing to engage with its values and principles. It has promoted social justice for, and the citizenship and human rights of, individuals who have been disenfranchised by society while challenging practitioners to question how they understand and respond to distress. However, its implementation and central tenets warrant further investigation as some service users struggle to benefit from ‘Recovery’.
